# Clinical Distribution Characteristics and Identification for Significant Liver Inflammation of Patients in Chronic Hepatitis B with Indeterminate Phase

**DOI:** 10.1155/2023/7264601

**Published:** 2023-07-11

**Authors:** Shanshan Chen, Xuan Dai, Yueyue Zhao, Jie Li, Xuehan Zou, Haijun Huang

**Affiliations:** ^1^Emergency and Critical Care Center, Department of Emergency Medicine, Zhejiang Provincial People's Hospital (Affiliated People's Hospital, Hangzhou Medical College), Zhejiang, Hangzhou 310014, China; ^2^Center for General Practice Medicine, Department of Infectious Disease, Zhejiang Provincial People's Hospital (Affiliated People's Hospital, Hangzhou Medical College), Zhejiang, Hangzhou 310014, China

## Abstract

**Aim:**

In clinical practice, a considerable proportion of patients with chronic hepatitis B (CHB) who do not conform to any immune status are considered to be in the “indeterminate phase”. In this study, we aim to study the clinical distribution characteristics and identification of significant liver inflammation in patients in indeterminate phase.

**Methods:**

This study retrospectively analyze clinical data of 1226 patients with CHB at two medical centers in Zhejiang province. According to American Association for the Study of Liver Diseases (AASLD) 2018 hepatitis B guidance, CHB can be divided into four phases: immune-tolerant phase, HBeAg-positive immune active phase, inactive phase, and HBeAg-negative immune active phase. Liver inflammation grade was evaluated using the Scheuer scoring system, and significant liver inflammation was defined as *G* ≥ 2.

**Results:**

The distribution of different immune status was as follows: 259 (21.1%) patients in immune-tolerant phase, 365 (29.8%) patients in HBeAg-positive immune active phase, 128 (10.4%) patients in inactive phase, and 33 (2.7%) patients in HBeAg-negative immune active phase. However, 441 (36.0%) patients did not meet any of the above immune phases, which were defined as indeterminate phase. Significant liver inflammation (54.1%) was common in CHB patients with indeterminate phase. Prothrombin time (PT), platelet count (PLT), alanine aminotransferase (ALT), and hepatitis B virus (HBV)-DNA were associated with significant inflammation.

**Conclusions:**

The results of this study showed that about 36.0% of patients were divided into indeterminate phase. The proportion of patients with significant inflammation in indeterminate phase and liver inflammation becomes more severe with aggravation of fibrosis stage. PT, PLT, ALT, and HBV-DNA may have a significant correlation with severe inflammation and prognosis of CHB.

## 1. Introduction

Hepatitis B virus (HBV) infection remains a serious public health problem, presenting a global epidemic, with approximately 1 million patients dying each year from chronic HBV-related complications [1–3]. According to the WHO, approximately 2 billion people worldwide have been infected with HBV, of which more than 240 million are chronically infected with HBV [4]. Approximately, 15%–40% of patients develop cirrhosis, liver decompensation, and hepatocellular carcinoma (HCC) [2, 3]. Thus, early identification of chronic hepatitis B (CHB) histology and administration of antiviral therapy can prevent further disease progression [5].

The natural history of chronic HBV infection is a complex and dynamic process, which is mainly determined by the interaction of virus, host, and environment [6, 7]. Therefore, individualized management of the pathogenesis with CHB in clinical practice is crucial. According to American Association for the Study of Liver Diseases (AASLD) 2018 hepatitis B guidance, Asian Pacific Society of Hepatology (APASL), and European Society of Hepatology (EASL) guidelines for diagnosis and treatment of CHB [1, 5, 8], chronic HBV infection can be classified into four phases: immune-tolerant phase, HBeAg-positive immune active phase, inactive phase, and HBeAg-negative immune active phase. Judging the different immune status of CHB patients is helpful for evaluating the prognosis with disease and making early treatment decisions [9]. However, the condition of CHB patients was more complex, many patients with increased ALT but low HBV-DNA, or increased HBV-DNA but low ALT levels, and these patients do not fit into the diagnostic criteria of any of above phases. Thus, this unclear state was defined as “indeterminate phase” [10]. According to current clinical guidelines, the management of patients with CHB of indeterminate phase recommends dynamic monitoring of serum ALT and HBV-DNA levels or evaluation of liver histology to determine the severity of CHB, and antiviral therapy is generally not recommended for these patients [1, 5, 11]. However, some studies have observed that untreated with indeterminate phase patients have higher clinical risk than treated patients with active CHB [12–15]. Therefore, there is controversy regarding the management of indeterminate phase CHB patients. However, there are few studies on the clinical distribution characteristics of indeterminate phase patients and the risk factors affecting significant liver inflammation in indeterminate phase patients.

Therefore, this study aims to retrospectively analyze clinical distribution characteristics and early identification of significant liver inflammation with indeterminate phase patients, which is of great significance for early antiviral treatment decision-making and disease prognosis.

## 2. Methods

From January 2012 to August 2021, the medical records of 1226 patients diagnosed with CHB who underwent liver biopsy were analyzed retrospectively at Zhejiang Provincial People's Hospital (Zhejiang, China) and the First Affiliated Hospital of Zhejiang University (Zhejiang, China). Chronic HBV infection was defined as hepatitis B surface antigen (HBsAg) positivity for at least 6 months [3].

The inclusion criteria were confirmed CHB infection. The exclusion criteria included: (1) hepatitis C virus (HCV) infection, hepatitis D virus (HDV) infection, co-infection with human immunodeficiency virus (HIV); (2) another cause of chronic liver disease, alcoholic liver disease, non-alcoholic fatty liver disease (NAFLD), autoimmune liver disease, decompensated cirrhosis, HCC; (3) incomplete clinical laboratory data, an inadequate liver biopsy sample; and (4) patients who underwent liver transplantation before enrollment. The retrospective study was approved by the Ethics Committee of Zhejiang Provincial People's Hospital and the First Affiliated Hospital of Zhejiang University.

### 2.1. Liver Biopsy

All patients underwent ultrasound-guided percutaneous liver biopsy. Liver biopsy was performed with an 18G biopsy needle. Biopsy specimens were formalin-fixed, embedded in paraffin, and stained with hematoxylin and eosin (HE). Each specimen was required to be at least 1.5 cm in length and contain at least six complete portal tracts. Histological necro-inflammation grade (G0–4) and fibrosis stage (S0–4) were evaluated according to the Scheuer classification system [16]. All specimens were independently evaluated by two pathologists who were blinded to patient's clinical data. Depending on the histology changes, G0–1 was considered as no or mild inflammation, G2–4 was considered as significant inflammation, and significant fibrosis was defined as S2–4 [16–19].

### 2.2. Data Collection

Clinical and laboratory parameters of patients were retrospectively collected from electronic medical records, including age, gender, white blood cell (WBC), platelet count (PLT), prothrombin time (PT), international normalized ratio (INR), albumin (ALB), globulin (GLB), aspartate aminotransferase (AST), alanine aminotransferase (ALT), alkaline phosphatase (ALP), glutamyl transpeptidase (GGT), serum total bilirubin (TBIL), HBsAg, and hepatitis B surface E antigen (HBeAg) status. Core antibody (anti-HBC) was detected using the CLIA system. The serum load of HBV-DNA was assessed via real-time polymerase chain reaction (ABI 7300 platform, Applied Biosystems, Foster City, CA, USA). Blood tests of the cohort were obtained on the day before liver biopsy.

### 2.3. Statistical Analysis

The data were analyzed using SPSS software ver. 25.0 (SPSS Inc./IBM, Chicago, IL, USA) and GraphPad Prism ver. 8.0.1 software. Continuous quantitative variables are expressed as the median (interquartile ranges [IQR]), and categorical variables are expressed as numbers or percentages. Comparisons between groups were made between continuous and categorical variables using Pearson chi-square test for categorical variables and Kruskal–Wallis test for continuous variables. To identify predictors of significant liver inflammation in the indeterminate phase, univariate and multivariate logistic regression analyses were used to determine. The area under the receiver operating characteristic (AUROC) curve was used to analyze and evaluate. The cut-off values were determined by Youden's index, which was the optimal combination of sensitivity and specificity. *P* < 0.05 was statistically significant.

## 3. Results

### 3.1. Clinical Distribution Characteristics of CHB Patients with Different Immune Status

**Figure 1:**
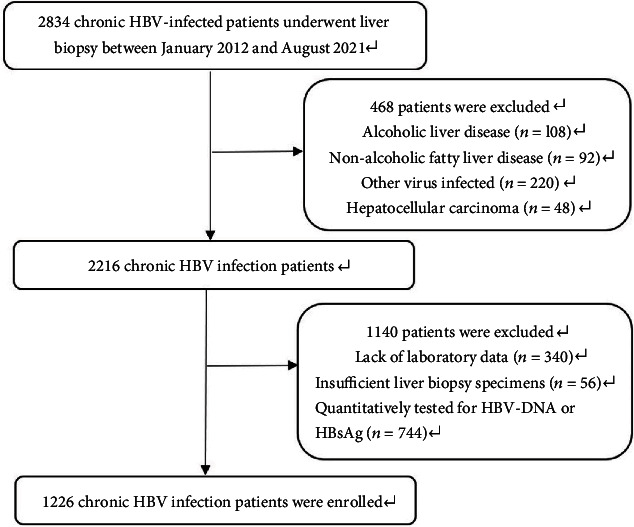
The flow chart of the study population. HBV, hepatitis B virus.

**Figure 2:**
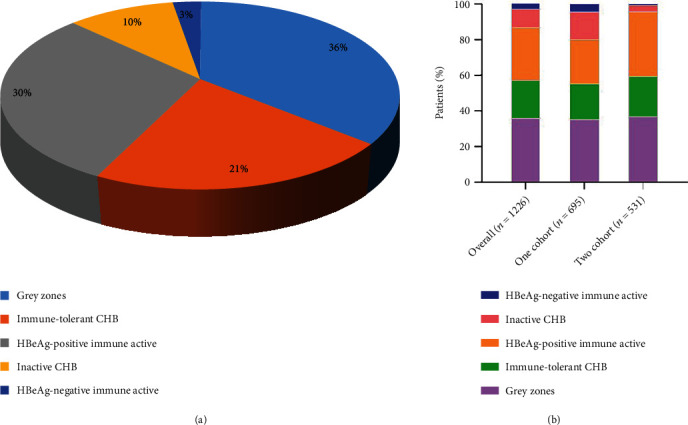
(a) The distribution of different immune status in all CHB patients. (b) Distribution of CHB patients with overall, one cohort and two cohort; one cohort: Zhejiang Provincial People's Hospital; two cohort: The First Affiliated Hospital of Zhejiang University.

A total of 2834 CHB patients who underwent liver biopsy were included in this study. Based on the exclusion criteria, 468 patients were excluded. In addition, 340 patients who were lack of laboratory data and 56 patients with insufficient liver biopsy specimens were excluded. A total of 744 patients who were not quantitatively tested for HBV-DNA or HBsAg were also excluded. Finally, a total of 1226 CHB patients were included in this study for analysis.  Figure 1 shows the flow chart of patient exclusion. In all patients, the distribution of different immune status was as follows: 259 (21.1%) patients in immune-tolerant phase, 365 (29.8%) patients in HBeAg-positive immune active phase, 128 (10.4%) patients in inactive phase, and 33 (2.7%) patients in HBeAg-negative immune active phase. In addition, a total of 441 (36.0%) patients at two medical centers did not fit into any immune status and were considered to be in the indeterminate phase (Figure 2(a)). The number of patients with indeterminate phase HBV infection in the medical centers was 35.4% in Zhejiang Provincial People's Hospital and 36.7% in the First Affiliated Hospital of Zhejiang University (Figure 2(b)).

### 3.2. Clinical Baseline Characteristics of CHB Patients with Different Immune Status

Table 1 describes the demographic and clinical laboratory indicators of CHB patients with different immune states. The median age of patients with different immune states was different, and the median age of patients with inactive phase and HBeAg-negative immune active phase was significantly higher than that of patients with immune-tolerant phase and HBeAg-positive immune active phase (*P* < 0.001). The median age of patients was 41.0 (34.0–48.0) in indeterminate phase. 499 (63.6%) patients were male, and 284 (56.2%) patients were male in indeterminate phase. Male patients in HBeAg-positive immune active phase and HBeAg-negative immune active phase were higher than those in immune-tolerant phase and inactive phase. The median serum HBV-DNA level was 7.9 (Log10 IU/ml) in the immune-tolerant phase, which was higher than that in other immune states. The median HBsAg level of 3.4 (Log10 IU/ml), serum ALT level of 71 (IU/L), and AST level of 44 (IU/L) were the highest in the HBeAg-positive immune active phase. The PLT count of CHB patients in HBeAg-negative immune active phase was the lowest (*P* < 0.001). The median level of serum TBIL in HBeAg-negative immune active phase was 15.7 (IU/L), which was higher than that in other immune states (*P* = 0.002).

### 3.3. Distribution Characteristics of Hepatic Inflammation and Fibrosis in Different Immune Status

**Figure 3:**
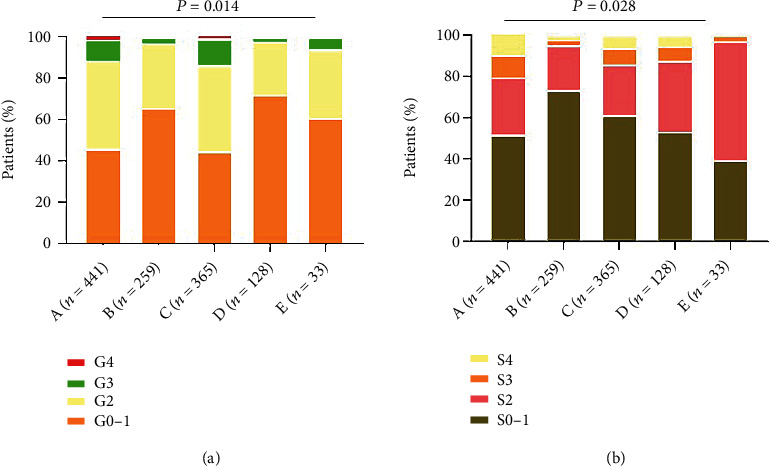
(a) The severity of liver inflammation grade with different immune states; A: indeterminate phase; B: immune-tolerant phase; C: HBeAg-positive immune active phase; D: inactive phase; E: HBeAg-negative immune active phase. (b) The severity of liver fibrosis stage with different immune states; A: indeterminate phase; B: immune-tolerant phase; C: HBeAg-positive immune active phase; D: inactive phase; E: HBeAg-negative immune active phase.

Effect of different immune states on the severity of liver inflammation and fibrosis stage. The proportion of mild liver inflammation was higher in immune-tolerant phase (65.6%) and inactive phase (71.9%). There were more patients with significant inflammation in the HBeAg-positive immune active phase (55.3%) and the HBeAg-negative immune active phase (39.4%), and there was a statistical difference between the groups (*P* = 0.014). In addition, significant liver inflammation (*G* ≥ 2) was observed in 54.1% of the patients during the indeterminate phase, 11.6% of the patients had a degree of inflammation of *G* ≥ 3, which was higher than that in the immune-tolerant phase, inactive phase, and HBeAg-negative immune active phases (Figure 3(a)). The proportion of mild fibrosis was higher in immune-tolerant phase (73.4%) and HBeAg-positive immune active phase (61.1%). There were more patients with significant liver inflammation in inactive phase (46.9%) and HBeAg-negative immune active phase (60.6%), and there was a statistical difference between the groups (*P* = 0.028). In addition, 48.3% of the patients had significant fibrosis (*S* ≥ 2) during the indeterminate phase, and 20.7% of the patients had fibrosis stage *S* ≥ 3, which was higher than that in the immune-tolerant phase, inactive phase, and HBeAg-negative immune active phase (Figure 3(b)).

### 3.4. The Relationship between Liver Inflammation and Liver Fibrosis in the Indeterminate Phase

**Figure 4:**
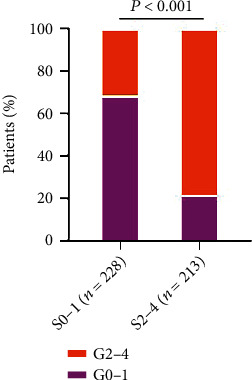
The relationship between liver inflammation grade and liver fibrosis stage in the indeterminate phase.

The severity of liver inflammation was significantly associated with liver fibrosis in patients with indeterminate phase CHB phase (*P* < 0.001). With the increase of liver fibrosis stage, the severity grade of liver inflammation also increased. In the *S* < 2 group, the proportion of patients with G0-1 was significantly higher than that in the *S* ≥ 2 group (68.4% vs. 21.6%). In the *S* ≥ 2 group, the proportion of patients with G2-4 was significantly higher than that in the *S* < 2 group (78.4% vs. 31.6%) (Figure 4).

### 3.5. Baseline Characteristics of Patients with Liver Mild and Significant Inflammation in the Indeterminate Phase

Among 441 patients with indeterminate phase CHB, 202 (45.8%) had mild inflammation (G0–1) and 239 (54.2%) had moderate-to-severe inflammation (G2–4). The median age in the significant inflammation group was 42.0 (33.0–49.0), which was higher than that in the mild inflammation group 40.0 (34.0–47.0). The proportion of male patients was higher, 67.8% patients in the significant group and 60.4% in the mild group. The serum ALT levels in the significant group and mild group were 40.0 (26.0–63.0) and 27.0 (19.0–47.3), respectively (*P* < 0.001). The levels of HBV-DNA in significant group and mild group were 4.5 (3.9–5.5) and 4.2 (3.6–4.9), respectively (*P* = 0.002). The levels of PT, INR, ALB, GLB, ALT, AST, GGT, and HBV-DNA in patients with significant inflammation were higher than those in patients with mild inflammation group (*P* < 0.001). The level of PLT in the significant inflammation group was lower than that in the mild inflammation group (*P* < 0.001). In moderate-to-severe inflammation group, 167 (69.9%) patients had significant fibrosis (S2–4), which was significantly higher than that in the mild inflammation group (G0–1) 46 (22.8%) patients (*P* < 0.001) (Table 2).

### 3.6. Univariate and Multivariate Analyses Were Performed to Analyze the Clinical Indicators Related to Indeterminate Phase Liver Inflammation

Univariate logistic regression analysis showed that the odds ratio (OR) of PT 1.756, 95% CI: (1.383–2.230), *P* < 0.001; the OR of PLT 0.994, 95% CI: (0.990–0.998), *P* < 0.001; the OR of ALB 0.948, 95% CI: (0.910–0.987), *P* = 0.01; the OR of ALT 1.008, 95% CI: (1.003–1.014), *P* = 0.003; the OR of AST 1.011, 95% CI: (1.002–1.019), *P* = 0.014; the OR of GGT 1.011, 95% CI: (1.003–1.018), *P* = 0.005; the OR of ALP 1.010, 95% CI: (1.002–1.017), *P* = 0.01; and the OR of HBV-DNA 1.332, 95% CI: (1.123–1.579), *P* = 0.001, which was associated with significant liver inflammation.

Multivariate analysis showed that the OR of PT 1.559, 95% CI: (1.210–2.009), *P* = 0.001; the OR of PLT 0.996, 95% CI: (0.992–1.000), *P* = 0.045; the OR of ALT 1.010, 95% CI: (1.002–1.018), *P* = 0.014; and the OR of HBV-DNA 1.262, 95% CI: (1.045–1.523), *P* = 0.016, which was an independent predictor of significant liver inflammation (Table 3).

### 3.7. Correlation between Independent Predictors and Liver Inflammation Activity Grade with GZ CHB Phase

**Figure 5:**
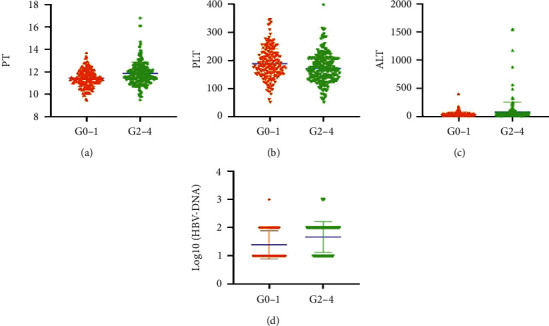
Correlation between independent predictors and liver inflammation activity grade with indeterminate phase CHB: (a) (PT), (b) (PLT), (c) (ALT), and (d) (HBV-DNA).

In CHB patients with indeterminate phase, PT, PLT, ALT, and HBV-DNA levels were significantly different between the moderate-to-severe liver inflammation group (G2-4) and the mild liver inflammation group (G0-1) (Figure 5). In patients with mild inflammation (G0-1), PT, ALT, and HBV-DNA levels were significantly lower than those in patients with moderate-to-severe inflammation (G2-4). The PLT level in the significant group was lower than that in the mild group.

**Figure 6:**
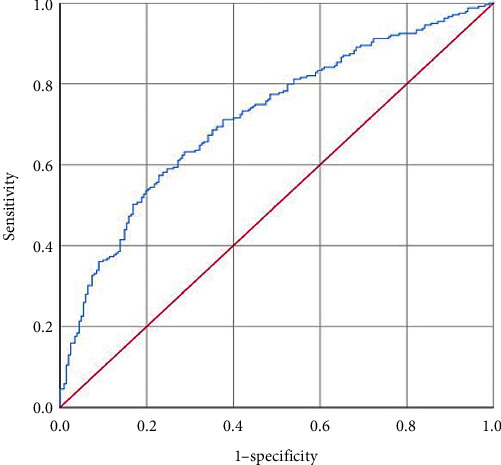
The ROC of model values for identifying moderate-to-severe inflammation in the indeterminate phase patients.

Based on PT, PLT, ALT, and HBV-DNA construct indicators, the AUROC was 0.717 (SE, 0.024, 95% CI: 0.669–0.764, *P* < 0.001). The sensitivity was 63.2%, and the specificity was 71.3% (Figure 6), which had a better diagnostic value for evaluating significant inflammation.

## 4. Discussion

Assessment of immune states and early identification of the severity of liver inflammation were critical for antiviral treatment decisions and disease prognosis in patients with CHB [1, 5]. In this study, we analyzed the clinical distribution characteristics of different immune status in patients with CHB. Analysis revealed that 36.0% of the CHB patients did not conform to any immune status and were considered to be in the indeterminate phase.

At present, the severity of liver injury, antiviral treatment decision, and long-term prognosis of patients with indeterminate phase CHB are controversial. Studies have reported that high levels of HBV-DNA and ALT levels are considered risk factors for significant liver histology in patients with CHB [20–22]. A multicenter retrospective study of the United States showed that among 3366 CHB patients enrolled, approximately, 38.7% of chronic HBV-infected patients in the indeterminate phase [10], which is consistent with our study. Yao et al. reported on 4759 patients of HBV infection in Nanjing, China, showing that 27.78% of the patients were in the indeterminate phase [23]. Spradling et al. followed up 1598 patients with chronic HBV infection in the United States, with a median follow-up time of 6.3 years, and the results showed that about 55% of patients were in the indeterminate phase [24]. A total of 327 HBV-infected patients with HBeAg-negative, ALT level continuous normal (≤40 IU/L), and relatively low level of HBV-DNA were analyzed by liver biopsy pathology. The number of patients with indeterminate phase was approximately 59.0% [25]. Therefore, regular follow-up and dynamic monitoring of patients' HBV-DNA and ALT levels are essential for the formulation of reasonable antiviral therapy and the prognosis of the disease.

For chronic HBV infection, the development of the disease is a dynamic process, and the infection status also exists for a long time. Therefore, for chronic HBV-infected patients, follow-up management and individualized management are needed to identify the severity of liver histology at early stage to prevent the progression of the disease. In an American study, 1465 patients with chronic HBV infection were followed up. The cumulative incidence of HCC in immune-tolerant, active, inactive, and indeterminate phase CHB patients were 0 (0/10), 21% (96/457), 3% (3/112), and 9% (80/886), respectively. Patients with indeterminate phase CHB were significantly higher than those with immune-tolerant and inactive phase [24].

It is not uncommon for CHB patients with normal or slightly elevated ALT levels to have moderate and severe inflammation. Recently, more evidences have shown that CHB patients with persistent normal ALT levels have significant liver histological changes, even in some patients with low viral load which indicates that inactive carries may have risk of disease progression such as cirrhosis and HCC [26, 27]. Besides, studies have reported that 27.1% of patients with normal ALT and low HBV-DNA levels had liver necro-inflammation ≥G2 [26]. Even if ALT is normal and there is no liver fibrosis, there is obvious inflammation in 28.7% of patients [28]. Similarly, our study found significant differences in the distribution characteristics of inflammation grade and fibrosis stage among different immune states. Moderate-to-severe liver inflammation (*G* ≥ 2) occurred in more than 50% of the patients in the indeterminate phase, which was significantly higher than that of the immune status. Similarly, *S* ≥ 3 has the highest proportion of patients in the indeterminate phase. The patients with *S* ≥ 2 were significantly higher than those in immune-tolerant phase, immune active phase, and inactive phases. The proportions of *G* ≥ 3 and *S* ≥ 3 were higher the indeterminate phase and immune activity phase. For patients with chronic HBV infection in the indeterminate phase, the results of examination alone may not be able to accurately assess the natural history stage, so dynamic follow-up observation is needed.

We further analyzed the risk factors of CHB in the indeterminate phase. PT, PLT, ALT, and HBV-DNA are independent risk factors for significant liver inflammation in patients with indeterminate phase CHB. Based on the above indexes, the Area Under Curve (AUC) of the combined model was 0.717, and its sensitivity (63.2%) and specificity (71.3%) were high, which had a better diagnostic value for the diagnosis of significant liver inflammation.

PT represents the hepatocyte synthetic function and is associated with the prognosis of significant liver inflammation [29]. With the increase of liver inflammation grade, PT level increased, and the level of group G2–4 was significantly higher than that of group G0–1. There was a significant negative correlation between PLT count and liver inflammation. PLT level decreased with the increase of inflammation grade. The reason for the reduced PLT count in patients with significant liver inflammation is not clear and may be related to concomitant fibrosis [30]. In clinical practice, serum ALT levels have been considered the most common biomarkers of liver inflammation activity and are widely used to assess the severity of liver necrotizing inflammation [31]. With the increase of liver inflammation grade, ALT level increased, and the level of group G2–4 was higher than that of group G0–1. Our study showed that serum HBV-DNA levels in patients with significant inflammation group were significantly higher in patients with mild inflammation. Elevated serum HBV-DNA levels are a risk factor for significant liver inflammation in patients with CHB, which is consistent with the study by Wu et al. [30]. HBV may cause persistent or episodic immune-mediated inflammation in hepatocytes [32]. Other studies have reported that elevated serum HBV-DNA levels are closely related to the occurrence of HCC [33, 34]. Therefore, antiviral therapy should be considered for patients with HBV infection with elevated HBV and significant liver inflammation, regardless of ALT level.

Our study has some limitations. First of all, this is a retrospective study with data from two medical centers, which may cause selection bias. Moreover, we did not analyze the HBV genotype. Subsequent validation should be performed on more patients from different medical centers. Second, our assessment of liver inflammation in the indeterminate phase has not been compared with other methods, and the value of combining other methods needs to be further studied. Finally, the immune status and prognosis of CHB patients in the indeterminate phase have not been further studied.

In conclusion, our data show that approximately 36.0% of CHB patients are in the indeterminate phase. More than half of the patients in the indeterminate phase had significant liver inflammation. More importantly, a small percentage of patients have the possibility for cirrhosis. Therefore, regular follow-up of these related risk factors for indeterminate phase patients is very necessary for the subsequent long-term prognosis and antiviral therapy.

## Figures and Tables

**Table 1:** Clinical distribution characteristics of CHB patients with different immune states.

Variables	Immune active					*P* value
	Immune tolerant (*N* = 259)	HBeAg positive (*N* = 365)	Inactive (*N* = 128)	HBeAg negative (*N* = 33)	Indeterminate phase (*N* = 441)	
Age (year)	32.0 (27.0–40.0)	30.0 (26.0–37.0)	42.0 (34.0–49.0)	41.0 (37.0–47.0)	41.0 (34.0–48.0)	<0.001
Sex (male%)	135 (52.1%)	260 (71.2%)	76 (59.4%)	28 (84.8%)	284 (56.2%)	<0.001
WBC (×10^9^/L)	5.7 (4.7–6.9)	5.4 (4.6–6.4)	5.7 (4.6–6.4)	5.8 (4.5–6.9)	5.4 (4.5–6.6)	0.241
PLT (×10^9^/L)	198.0 (170.0–228.0)	189.0 (154.5–223.0)	189.0 (147.3–223.0)	182.0 (133.0–222.5)	174.0 (144.0–210.0)	<0.001
PT (s)	11.6 (11.1–12.1)	11.6 (11.1–12.2)	11.4 (11.0–12.0)	11.4 (10.8–11.8)	11.6 (11.1–12.2)	0.025
INR	1.0 (1.0–1.1)	1.0 (1.0–1.1)	1.0 (1.0–1.1)	1.0 (0.9–1.0)	1.0 (1.0–1.1)	0.002
ALB (g/L)	45.0 (42.3–47.5)	45.0 (42.2–47.5)	44.8 (43.2–46.7)	45.3 (43.1–48.1)	44.8 (42.2–47.3)	0.766
GLB (g/L)	28.4 (25.3–30.6)	28.5 (25.7–31.3)	28.0 (28.8–31.0)	28.2 (26.5–30.7)	28.1 (25.5–31.6)	0.875
ALT (U/L)	26.0 (19.0–32.0)	71.0 (53.0–111.0)	21.5 (15.3–30.5)	55.0 (47.0–86.5)	33.0 (22.0–54.0)	<0.001
AST (U/L)	24.0 (20.0–28.0)	44.0 (35.0–65.5)	23.0 (19.0–28.0)	36.0 (31.0–59.5)	29.0 (23.0–39.0)	<0.001
GGT (U/L)	16.0 (12.0–22.0)	30.0 (20.0–49.0)	17.5 (13.0–30.0)	36.0 (23.5–56.0)	23.0 (15.0–35.5)	<0.001
ALP (U/L)	68.0 (57.0–83.0)	78.0 (64.0–93.0)	71.5 (60.0–85.3)	84.0 (74.0–95.0)	78.0 (62.0–96.0)	<0.001
TBIL (*μ*mol/L)	13.0 (9.2–16.6)	14.0 (11.0–18.2)	15.3 (11.8–18.1)	15.7 (13.0–18.0)	14.0 (10.8–18.2)	0.002
Log10 (HBV-DNA IU/ml)	7.9 (7.3–8.4)	7.5 (6.7–8.2)	2.8 (2.4–3.1)	2.6 (2.3–3.1)	4.3 (3.7–5.3)	<0.001
Log10 (HBsAg IU/ml)	2.4 (2.4–4.7)	3.4 (2.4–4.5)	2.4 (2.3–2.4)	2.4 (1.9–2.4)	2.4 (2.4–3.3)	<0.001
Anti-HBC (S/CO)	8.9 ± 2.7	10.7 ± 2.4	10.3 ± 1.7	10.5 ± 1.5	11.2 ± 2.6	<0.001
Liver inflammation (%)						
0	4 (1.5)	1 (0.3)	2 (1.6)	2 (6.1)	2 (0.5)	<0.001
1	166 (64.1)	162 (44.4)	90 (70.3)	18 (54.5)	200 (45.4)	
2	81 (31.3)	152 (41.6)	33 (25.8)	11 (33.3)	188 (42.6)	
3	8 (3.1)	47 (12.9)	3 (2.3)	2 (6.1)	45 (10.2)	
4	0 (0)	3 (0.8)	0 (0)	0 (0)	6 (1.4)	
Fibrosis (%)						
0	69 (26.6)	49 (13.4)	13 (10.4)	2 (6.1)	67 (15.2)	<0.001
1	121 (46.7)	174 (47.7)	55 (44.0)	11 (33.3)	161 (36.5)	
2	56 (21.6)	90 (24.7)	44 (35.2)	19 (57.6)	122 (27.7)	
3	7 (2.7)	29 (7.9)	9 (7.2)	1 (3.0)	48 (10.9)	
4	6 (2.3)	23 (6.3)	7 (5.6)	0 (0)	43 (9.7)	

**Table 2:** Baseline characteristics of patients with liver mild and significant inflammation in the indeterminate phase.

Variables	G0–1 (*n* = 202)	G2–4 (*n* = 239)	*P* value
Age (year)	40.0 (34.0–47.0)	42.0 (33.0–49.0)	0.223
Sex (male%)	122 (60.4)	162 (67.8)	0.107
WBC (×10^9^/L)	5.4 (4.5–6.5)	5.4 (4.5–6.6)	0.895
PLT (×10^9^/L)	181.0 (153.0–220.3)	166.0 (138.0–206.0)	0.001
PT (s)	11.4 (11.0–11.9)	11.8 (11.2–12.3)	<0.001
INR	1.0 (1.0–1.1)	1.0 (1.0–1.1)	0.002
ALB (g/L)	45.4 (42.7–48.1)	44.2 (41.8–46.6)	0.003
GLB (g/L)	27.6 (25.5–30.9)	28.6 (25.6–32.0)	0.045
ALT (U/L)	27.0 (19.0–47.3)	40.0 (26.0–63.0)	<0.001
AST (U/L)	26.0 (21.0–32.5)	34.0 (26.0–45.0)	<0.001
GGT (U/L)	19.5 (14.0–30.0)	25.0 (18.0–43.0)	<0.001
ALP (U/L)	75.5 (60.8–91.0)	81.0 (62.0–99.0)	0.052
TBIL (*μ*mol/L)	13.0 (10.0–17.2)	14.3 (11.0–18.9)	0.155
Log10 (HBV-DNA IU/ml)	4.2 (3.6–4.9)	4.5 (3.9–5.5)	0.002
Log10 (HBsAg IU/ml)	2.4 (2.4–3.3)	2.4 (2.4–3.3)	0.536
Anti-HBC (S/CO)	11.2 (9.5–12.3)	11.2 (9.8–12.5)	0.493
Fibrosis (%)			
0	56 (27.7)	11 (4.6)	<0.001
1	100 (49.5)	61 (25.5)	
2	38 (18.8)	84 (35.1)	
3	7 (3.5)	41 (17.2)	
4	1 (0.5)	42 (17.6)	

**Table 3:** Logistic regression analysis of clinical indexes associated with significant liver inflammation in the indeterminate phase.

Variables	Univariate analysis		Multivariate analysis	
	OR (95% CI)	*P* value	OR (95% CI)	*P* value
Age (year)	1.012 (0.993–1.032)	<0.001		
Sex (male%)	0.784 (0.477–1.289)	0.338		
WBC (×10^9^/L)	0.990 (0.878–1.115)	0.863		
PT	1.756 (1.383–2.230)	<0.001	1.559 (1.210–2.009)	0.001
INR	0.918 (0.699–1.205)	0.537		
PLT (×10^9^/L)	0.994 (0.990–0.998)	0.001	0.996 (0.992–1.000)	0.045
ALB (U/L)	0.948 (0.910–0.987)	0.010	1.052 (0.918–1.192)	0.141
GLB (U/L)	1.036 (0.997–1.077)	0.069		
ALT (U/L)	1.008 (1.003–1.014)	0.003	1.010 (1.002–1.018)	0.014
AST (U/L)	1.011 (1.002–1.019)	0.014	0.991 (0.980–1.002)	0.111
GGT (U/L)	1.011 (1.003–1.018)	0.005		
ALP (U/L)	1.010 (1.002–1.017)	0.010	1.006 (0.998–1.014)	0.136
TBIL (U/L)	1.008 (0.990–1.027)	0.383		
Log10 (HBV-DNA IU/ml)	1.332 (1.123–1.579)	0.001	1.262 (1.045–1.523)	0.016
Log10 (HBsAg IU/ml)	1.002 (0.769–1.305)	0.991		
Anti-HBC (S/CO)	1.047 (0.970–1.131)	0.235		

## Data Availability

The data that support the findings of this study are available from the corresponding author upon reasonable request.
